# X‐Linked Hereditary Hydrocephalus Diagnosed in the Fetal Period With Adducted Thumb and a Novel L1CAM Variant: A Case Report

**DOI:** 10.1002/ccr3.70792

**Published:** 2025-08-14

**Authors:** Ryosuke Horiuchi, Hiroshi Sato, Chinami Asai, Fumika Hamaguchi, Yu Takaishi, Kensuke Fujiwara, Yukari Atsumi, Yukiko Ando, Takahito Kawata, Kazuyo Kakui

**Affiliations:** ^1^ Department of Obstetrics and Gynecology Hyogo Prefectural Amagasaki General Medical Center Amagasaki Hyogo Japan; ^2^ Department of Clinical Genetics Hyogo Prefectural Amagasaki General Medical Center Amagasaki Hyogo Japan

**Keywords:** adducted thumb, genetic counseling, Hirschsprung's disease, L1CAM mutation, prenatal MRI, X‐linked hydrocephalus

## Abstract

X‐linked hereditary hydrocephalus (XLH) is a congenital form of hydrocephalus caused by variants in the L1CAM gene on the X chromosome. Diagnosis is often made prenatally via ultrasound or magnetic resonance imaging (MRI), but specific features such as adducted thumbs are subtle and easily missed. We report a case in which prenatal MRI at 34 weeks gestation revealed fetal hydrocephalus and an adducted thumb, suggestive of XLH. Postnatal genetic testing confirmed a previously unreported frameshift variant in the L1CAM gene, c.2248dup (p.Tyr750LeufsTer36). The male infant required neurosurgical intervention and was also diagnosed with Hirschsprung's disease. Genetic testing confirmed that the mother was a heterozygous carrier. In a subsequent pregnancy, non‐invasive prenatal testing (NIPT) predicted a female fetus with no hydrocephalus. This case highlights the importance of thorough imaging and genetic evaluation in suspected XLH, especially given the increasing discovery of novel pathogenic variants.

## Summary

X‐linked hereditary hydrocephalus (XLH) is a common cause of fetal hydrocephalus. L1CAM gene variants are causative, and novel pathogenic variants continue to emerge, making accurate diagnosis and variant evaluation increasingly important. This case highlights the importance of early genetic testing, as the identification of a novel L1CAM variant supported the prenatal diagnosis of X‐linked hydrocephalus and informed postnatal management.

## 1 Case History/Examination

**FIGURE 1:**
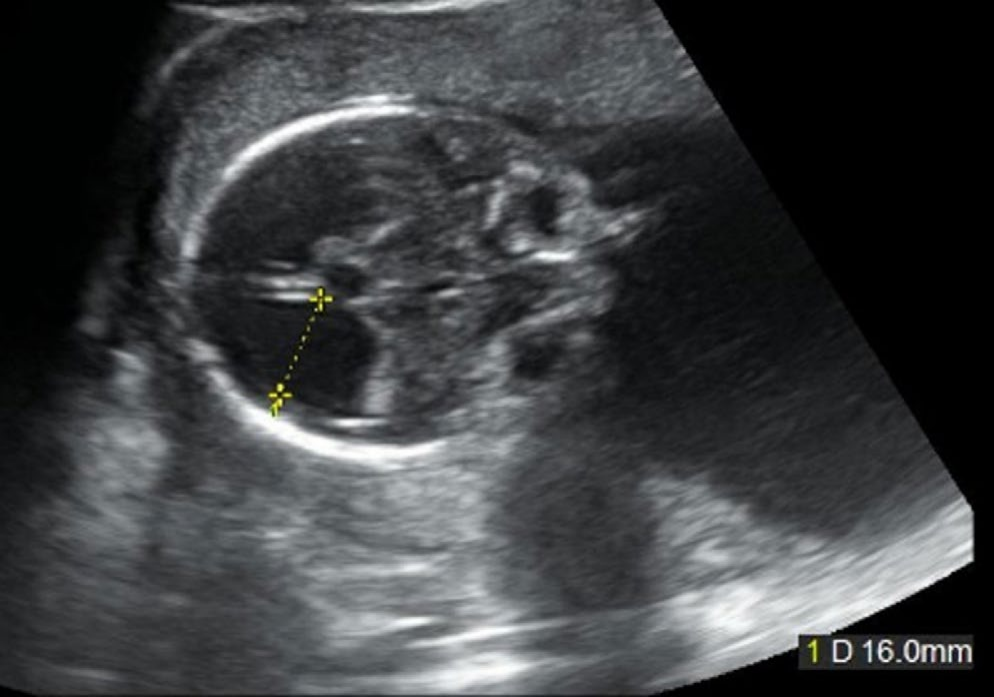
Ultrasonography at 20 weeks of gestation revealed an enlargement of the posterior horn of the lateral ventricle to 16 mm.

A 29‐year‐old Japanese woman, Gravida 1 Para 0, conceived with ovulation induction. Her medical and family history were unremarkable. Routine ultrasound at 20 weeks' gestation revealed enlargement of the fetal lateral ventricles (posterior horn 16 mm) (Figures 1). Follow‐up imaging showed progressive ventriculomegaly and cortical thinning, suggesting fetal hydrocephalus.

**FIGURE 2:**
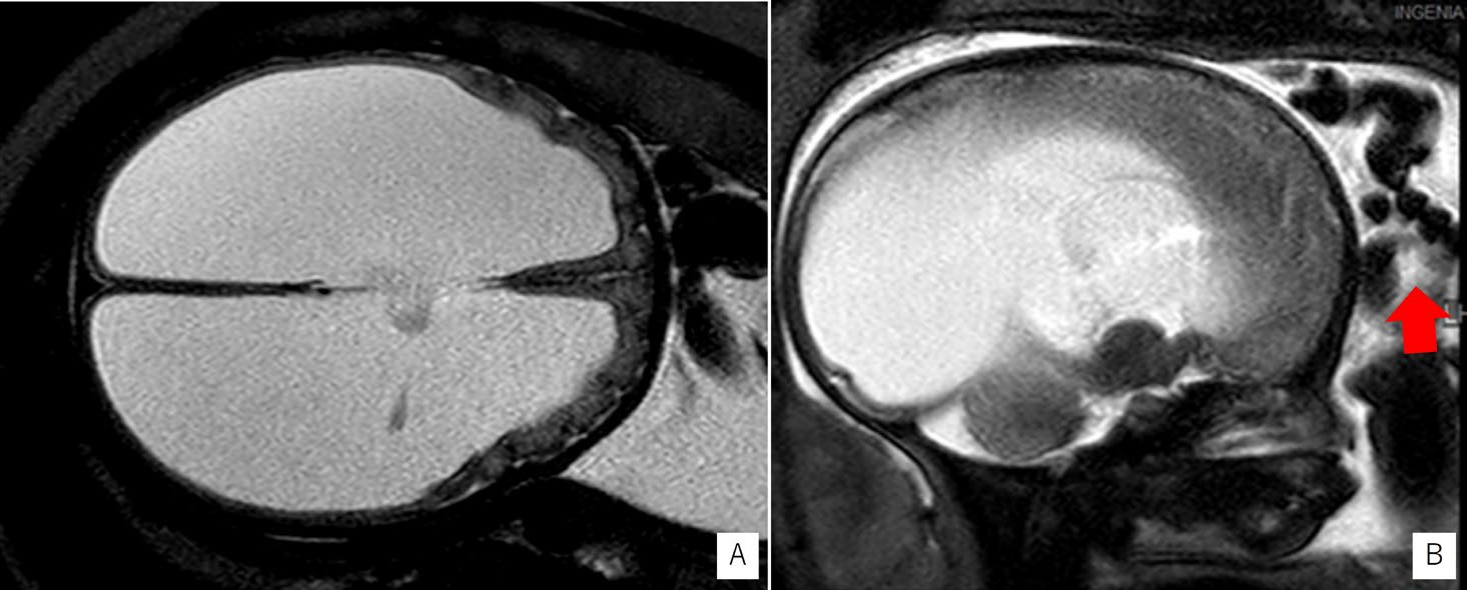
Fetal MRI images at 34 weeks of gestation. (A) Axial T2‐weighted image showing marked enlargement of the lateral ventricles. (B) Sagittal T2‐weighted image showing adduction and flexion of the left thumb (red arrow). T1‐weighted images were not acquired in this case.

MRI at 34 weeks gestation confirmed enlargement of the lateral and third ventricles, with suspected aqueductal stenosis (Figures 2A). An adducted left thumb was also observed (Figures 2B). The fetus was male. Given these findings and clinical suspicion of XLH, prenatal genetic testing was offered but declined.

## 2 Differential Diagnosis, Investigations and Treatment

Biparietal diameter was significantly enlarged (110.1 mm, +7.15 SD). A cesarean section was planned at 36 weeks 3 days due to breech presentation. A male infant was delivered with Apgar scores of 7 and 9 at 1 and 5 min, respectively. Birth weight was 3054 g (+1.94 SD), and head circumference was 44.2 cm (+9.5 SD).

**FIGURE 3:**
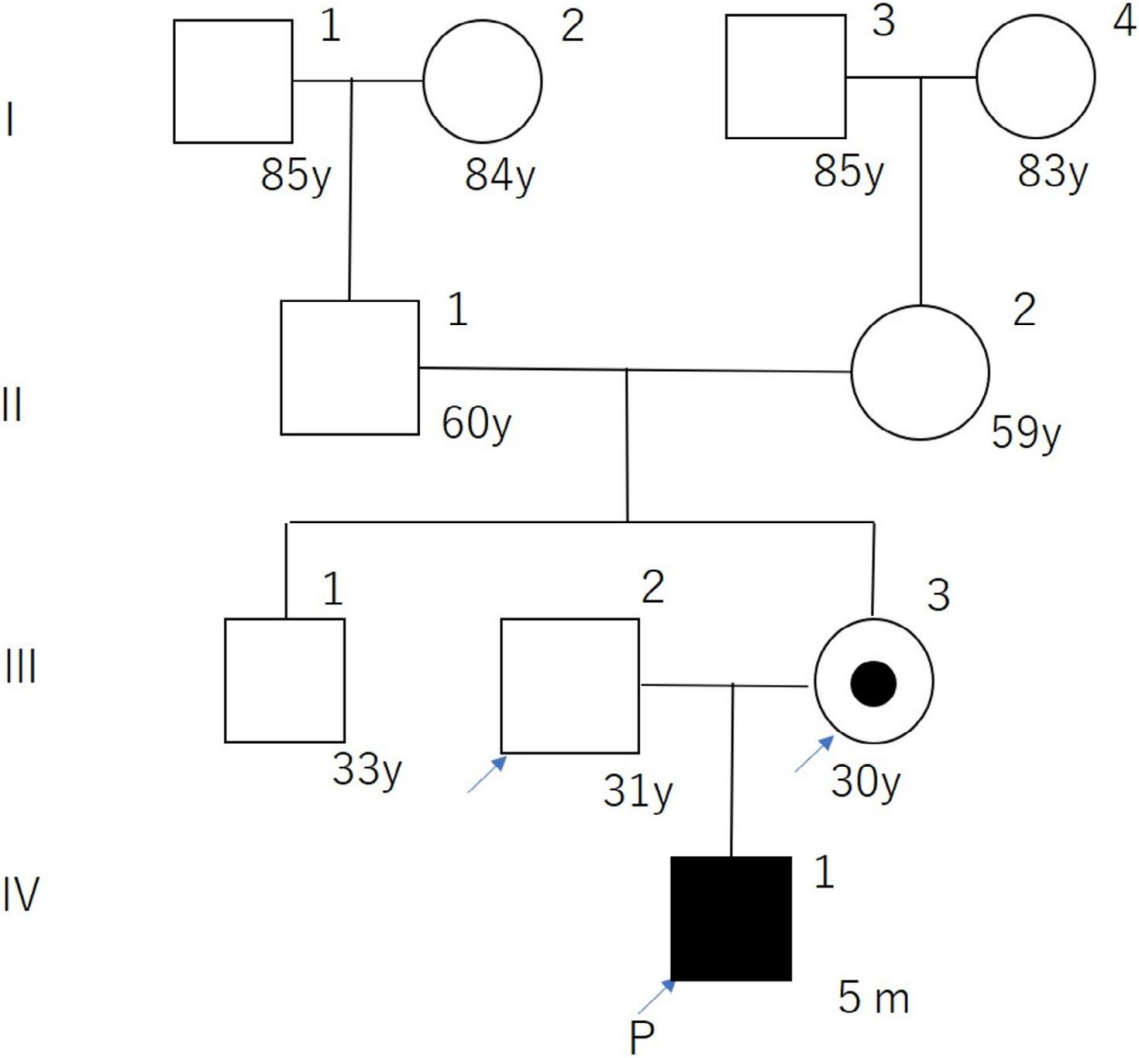
Pedigree of the patient and her family. Generations are shown in Roman numerals, individuals in Arabic numerals. Solid symbols indicate individuals diagnosed with XLH. The proband is marked by an arrow and the letter P.

**FIGURE 4:**
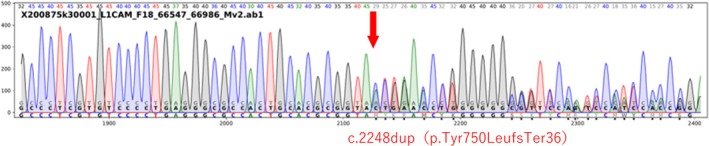
Sanger sequencing chromatogram of the L1CAM gene showing a duplication of a thymine at position c.2248 (c.2248dupT), resulting in a frameshift (p.Tyr750LeufsTer36). Although the base numbering is not shown in the chromatogram, the duplication was confirmed by alignment with the reference sequence (NM_000425.3), and the downstream sequence shift is consistent with a single‐base duplication.

He was admitted to the NICU. On day 2, elevated intracranial pressure necessitated cerebrospinal fluid reservoir placement. Hirschsprung's disease was suspected and later confirmed by surgery at 38 days. A ventriculoperitoneal shunt was placed at 48 days. After ventriculoperitoneal shunt placement, no further progression of hydrocephalus has been observed. However, the child presents with severe developmental delay and spastic paraplegia, and is currently under regular follow‐up at our hospital's pediatric department. Genetic testing showed a hemizygous c.2248dup (p.Tyr750LeufsTer36) variant in the L1CAM gene. We then conducted another genetic counseling session with the client (mother) and her husband and made a carrier diagnosis for the client. The client was found to be heterozygous for the same variant as the proband (baby) (Figures 3, 4).

## 3 Conclusion and Results

Two years later, the mother conceived naturally. Non‐invasive prenatal testing (NIPT) indicated a female fetus. Ultrasound showed no hydrocephalus. A healthy girl was born by repeat cesarean section at 38 weeks. Although carrier status was unknown, genetic counseling for future testing was planned upon her reaching adulthood.

## 4 Discussion

X‐linked hereditary hydrocephalus (XLH) was first reported in 1949 as a disease characterized by hydrocephalus caused by stenosis of the cerebral aqueduct, neuromotor retardation, spastic paralysis of the lower limbs, and adduction flexion of the thumb [1]. The L1CAM gene has been identified as the gene responsible for XLH. L1CAM is a neuronal cell adhesion molecule located on X chromosome q28 and has important functions in the development of the nervous system [2].

With recent improvements in the diagnostic accuracy of ultrasound, the incidence of fetal ventricular enlargement detected during antenatal screening has increased. Recent reports suggest that the incidence of fetal ventriculomegaly is around 1% and is generally defined as a diameter of 10 mm or greater in the posterior horn of the lateral ventricles, regardless of gestational age. Severe ventriculomegaly is more likely than mild ventriculomegaly to cause clinical symptoms such as neurodevelopmental delay. If ventriculomegaly is detected on fetal ultrasound, detailed intracranial assessment is required. MRI is useful for fetal intracranial assessment. MRI for detailed intracranial observation is particularly useful in cases with enlarged ventricles on ultrasound [3]. Adducted thumbs are reported to be an early sign of neurodevelopmental disorders [4]. Izumi et al. reported that adducted thumbs are present in only half of patients before 24 weeks' gestation, but they are present in most cases of XLH in the second trimester [5]. Therefore, the timing of evaluation needs to be considered. In our case, the diagnosis of adducted thumbs was made by MRI, but there are no previous reports of adducted thumbs diagnosed by MRI. A more detailed evaluation of the brain by ultrasound and MRI may provide more information.

The mode and timing of delivery should be discussed. Increased head circumference may complicate delivery, even by cesarean section, increase the risk of maternal injury, and adversely affect the child's prognosis and should be considered on a case‐by‐case basis. There is no established method or timing of delivery in cases of fetal hydrocephalus, but cesarean delivery after confirmation of lung maturity is considered appropriate [6]. Kuller JA et al. suggested that cesarean delivery of a hydrocephalic fetus with macrocephaly contributes to a better prognosis [7]. In our case, we did not perform tests to confirm lung maturity. Although the fetus was delivered prematurely at 36 weeks' gestation, it was delivered at a time when lung maturity was expected.

The molecular mechanism and structure of the L1CAM protein are well understood. It is a plasma membrane protein of 1238 amino acids, with an extracellular portion of 1101 amino acids containing 6 IgG domains and 5 fibronectin III domains. Yamasaki M et al. classified the proteins into three classes according to the nature of these variants [8]. The variant in our case is class 3, which is the most severe. Michaelis RC et al. reported that the fibronectin domain variant is more severe than the IgG domain variant [9]. The c.2248dupT variant identified in this case is a novel frameshift in the fibronectin type III‐2 domain of L1CAM, a region where multiple pathogenic variants have been reported. Given the predicted loss of function and its location, this variant is considered likely pathogenic (PVS1) [10].

This variant is located within the fibronectin III‐2 domain of L1CAM, where several pathogenic missense and frameshift variants have been previously reported, such as p.Val788Phe and p.Tyr784Cys [11, 12]. These observations support the pathogenic relevance of this domain [13, 14, 15].

The probability of a de novo mutation in an X‐linked recessive condition is typically estimated at one‐third, based on classic genetic counseling principles [16]. There were no other XLH cases in the family; the probability of a de novo mutation was estimated to be about 1/3. The probability of the client being a carrier was estimated to be about 2/3, which should be taken into account when diagnosing carriers of X‐linked diseases, as the woman will be screened to see if she is a carrier.

Regarding prenatal diagnosis, Serikawa T et al. reported that in families where the first child has XLH and the mother is known to be a carrier, the next and subsequent pregnancies should be sexed by chromosome analysis, followed by L1CAM gene analysis if the child is a boy [17]. As in this case, using NIPT to diagnose the sex of the fetus may be useful in avoiding invasive tests such as amniocentesis. Preimplantation genetic diagnosis (PGD) is limited to in vitro fertilization, but PGD can reduce the burden on the mother. We need to offer this couple options based on the latest evidence when they consider their next pregnancy.

## 5 Conclusion

This case highlights the importance of detailed fetal imaging in diagnosing XLH. MRI findings, particularly adducted thumbs, may raise early suspicion. Identification of novel L1CAM variants is vital for expanding our understanding of this condition. Genetic counseling and testing remain essential for informed family planning.

## Author Contributions

**Ryosuke Horiuchi:** investigation, writing – original draft. **Hiroshi Sato:** conceptualization, investigation, writing – original draft. **Chinami Asai:** investigation, writing – original draft. **Fumika Hamaguchi:** investigation. **Yu Takaishi:** investigation. **Kensuke Fujiwara:** data curation, investigation. **Yukari Atsumi:** data curation, investigation. **Yukiko Ando:** data curation, investigation. **Takahito Kawata:** data curation, investigation. **Kazuyo Kakui:** supervision, writing – review and editing.

## Consent

Written informed consent was obtained from the patient for publication of this case report and accompanying images.

## Conflicts of Interest

The authors declare no conflicts of interest.

## Data Availability

The clinical data presented in this case report are not publicly available due to patient privacy, but further details are available from the corresponding author upon reasonable request and with appropriate ethical approval.
